# New Method of Staining Micro-Organisms in the Blood

**Published:** 1895-02

**Authors:** 


### SELECTIONS.

### New Method of Staining Micro-Organisms in the Blood.

A communication on this subject was made by M. H. Vincent at the last meeting of the Societe de Biologie.

The process is applicable to every variety of micro-organism occurring in the blood, but is particularly serviceable in microscopical examination of bacteria which do not take coloration by the method of Gram, and of various parasites, such as hematozoa of pludism.

The process is based upon the following principle, viz., coloring matters fix themselves not to the protoplasm but to the hemoglobin itself. If, therefore, the latter color-taking constituent be made artificially to disappear and the coloring agent be then brought to bear, the blood globules which mask the bacteria become invisible and the microbes alone remain colored and stand out under the microscope with great clearness.

Among the different dissolvents tried; M. Vincent has selected the following liquid which does not alter the form of the globules and leaves no deposit and no striæ:

| | |
|---|---|
| Aqueous solution carbolic acid, 5 p. c., | 6 centim. cubes. |
| Water Saturated with Na Cl., | 30 " " |
| Glycerine, | 30 " " |
| Filter. | |

The blood spread in a thin layer (or in a thick layer when microbes rare in number, are being sought) is slowly dried either at ordinary temperature or a feeble heat. It is then treated with the fluid above described, which dissolves entirely the hemoglobin. At the end of from half a minute to two minutes the fluid is drained off, the blood washed with distilled water, and coloration is effected with carbolized methylene blue with the addition of from 1 to 20 per cent. of aqueous solution of methyl violet.—*Paris Cor. Med. Press and Circular.*

